# Analysis of the immune-inducible transcriptome from microbial stress resistant, rat-tailed maggots of the drone fly *Eristalis tenax*

**DOI:** 10.1186/1471-2164-8-326

**Published:** 2007-09-17

**Authors:** Boran Altincicek, Andreas Vilcinskas

**Affiliations:** 1 Institute of Phytopathology and Applied Zoology, Interdisciplinary Research Center, Justus-Liebig-University of Giessen, Heinrich-Buff-Ring 26-32, D-35392 Giessen, Germany

## Abstract

**Background:**

The saprophagous and coprophagous maggots of the drone fly *Eristalis tenax *(Insecta, Diptera) have evolved the unique ability to survive in aquatic habitats with extreme microbial stress such as drains, sewage pools, and farmyard liquid manure storage pits. Therefore, they represent suitable models for the investigation of trade-offs between the benefits resulting from colonization of habitats lacking predators, parasitoids, or competitors and the investment in immunity against microbial stress. In this study, we screened for genes in *E. tenax *that are induced upon septic injury. Suppression subtractive hybridization was performed to selectively amplify and identify cDNAs that are differentially expressed in response to injected crude bacterial endotoxin (LPS).

**Results:**

Untreated *E. tenax *maggots exhibit significant antibacterial activity in the hemolymph which strongly increases upon challenge with LPS. In order to identify effector molecules contributing to this microbial defense we constructed a subtractive cDNA library using RNA samples from untreated and LPS injected maggots. Analysis of 288 cDNAs revealed induced expression of 117 cDNAs corresponding to 30 novel gene clusters in *E. tenax*. Among these immune-inducible transcripts we found homologues of known genes from other Diptera such as *Drosophila *and *Anopheles *that mediate pathogen recognition (e.g. peptidoglycan recognition protein) or immune-related signaling (e.g. relish). As predicted, we determined a high diversity of novel putative antimicrobial peptides including one *E. tenax *defensin.

**Conclusion:**

We identified 30 novel genes of *E. tenax *that were induced in response to septic injury including novel putative antimicrobial peptides. Further analysis of these immune-related effector molecules from *Eristalis *may help to elucidate the interdependency of ecological adaptation and molecular evolution of the innate immunity in Diptera.

## Background

*Eristalis tenax *belongs to the hover flies, family Syrphidae (Diptera), with more than 5,000 described species, which occurred late in evolution and radiated in the Early Tertiary [1]. In contrast to the predominantly flower-feeding habitats of adult syrphids, their maggots are found in a very diverse array of habitats. Those of subfamily Eristalinae are saprophagous, coprophagous and aquatic filterfeeders. Combined analysis of molecular and morphological characters supports both frequent shifts between larval feeding habitats within the Eristalinae and the monophyly of this subfamily [2]. The rat-tailed maggots of *E. tenax *are characterized by their extended anal breathing tube that functions as a snorkel and enables survival in waters with anaerobic conditions. They realize a unique ecological niche by preferably living in stagnant aquatic environments with high organic and microbial contamination. Because of the preference of *E. tenax *larvae for dirty waters with anaerobic conditions, they are reliable and prominent indicators in the biological assessment of water quality for extremely high pollution with organic material [3]. They thus represent a suitable model to investigate the trade-offs between the benefits resulting from colonization of habitats lacking predators, parasitoids, or competitors and the investment in immunity against microbial stress.

The innate immunity of insects relies on immediate processes including cellular phagocytosis, encapsulation, hemolymph coagulation, and phenoloxidase activation leading to melanization. On the other hand sustainable defense is achieved by the massive synthesis of antimicrobial peptides like defensins and cecropins [4,5]. Insect immunity has been best analyzed in the fruit fly *D. melanogaster *[6,7], but results of comparative genome-wide analysis of immunity-related genes in *D. melanogaster*, *Anopheles gambiae*, and *Aedes aegypti *reveal that effector molecules from these dipteran species exhibit large variations [8]. For example, four defensin and four cecropin genes exist in *Anopheles*, making these more numerous than in *Drosophila *whereas some antimicrobial peptides that were identified in *Drosophila *are absent in *Anopheles *and *Aedes *[8]. The maggots of *E. tenax *have been recognized to be particularly resistant to microbial stress, because they are able to survive in aquatic habitats that are usually not colonized by other dipteran larvae or animals [3]. Hence we were interested to identify the immune-related genes of this insect.

To selectively identify immune-induced genes we used the subtractive suppression hybridization (SSH) method which amplifies differentially expressed cDNAs and simultaneously suppresses amplification of common cDNAs. This technique has been proven as a suitable tool for identification of immune-related genes in insects [9-11] and other invertebrates [12,13]. Here, we report analysis of the immune-inducible transcriptome of *E. tenax *maggots, collected from storage pits of liquid manure. This enabled identification of genes encoding novel proteins that are potentially involved in pathogen-recognition and immune signaling. In addition, we identified numerous potential antimicrobial peptides that most probably contribute to the observed immune defense that is obviously important to control the wide array of pathogens present in the habitat of *Eristalis *larvae.

## Results and discussion

### Subtracted cDNA library of immune challenged *E. tenax *larvae

**Figure 1:**
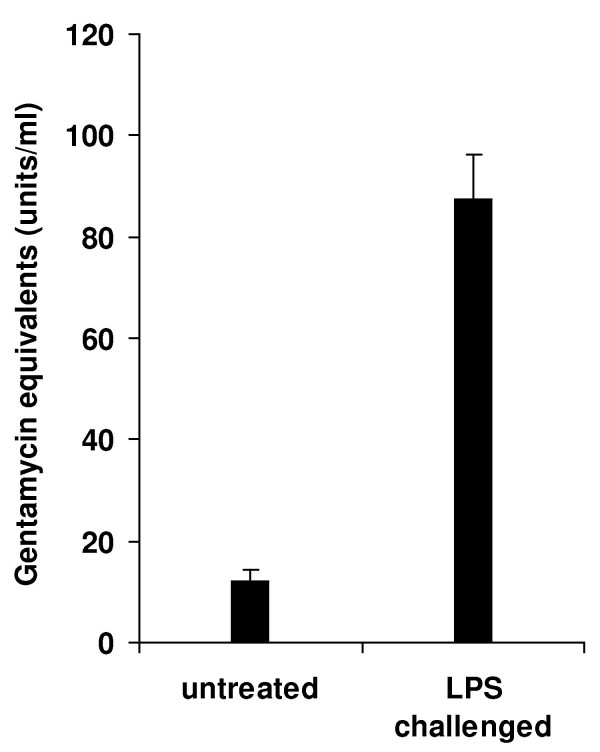
**Immune challenge of rat-tailed maggots of *E. tenax *induces the production of antimicrobial effector molecules**. Antibacterial activities represented by gentamycin equivalents of hemolymph from untreated and LPS injected larvae are shown. Results represent mean values of independent determinations ± S.D.

**Figure 2:**
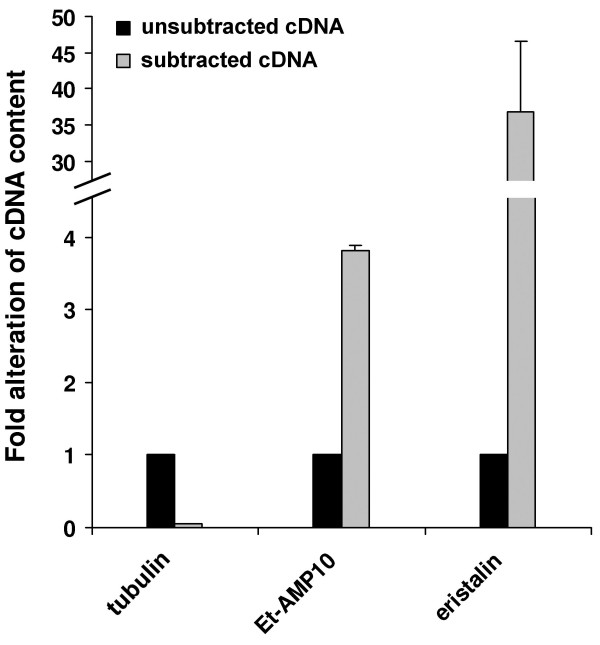
**Confirmation of the enrichment of immune-related transcripts in the subtracted cDNA library by quantitative real-time PCR analysis**. The relative amount of cDNAs of α-tubulin, Et-AMP10, and eristalin in the subtracted cDNA library (gray bars) is shown relative to their amount in unsubtracted cDNA library (black bars). The cDNA amount of the house-keeping gene α-tubulin was reduced about 18 fold by the subtraction procedure. In contrast, potentially immunity-related genes Et-AMP10 and eristalin were found to be 4 and 38 fold enriched in the subtracted cDNA library, respectively. Results represent mean values of three independent determinations ± S.D.

We observed a significant *E. coli *inhibitory activity of hemolymph samples from untreated *E. tenax *larvae that further increases upon immune challenge (Fig. 1). In order to construct a subtracted cDNA library enriched in immune-inducible genes we used purified RNA from LPS injected and untreated animals combined with a PCR-based SSH method. To induce strong and broad immune responses we injected a commercially available purified LPS preparation, which is known to contain impurities like nucleic acids, proteins and peptidoglycans, and which according to the supplier is commonly used as elicitor of immune responses in vertebrates and invertebrates. In order to confirm that the subtraction process has been performed efficiently, we analyzed the abundance of transcripts for the house-keeping gene α-tubulin and for two genes, the Et-AMP10 and eristalin. The latter ones were found in the present study to be induced in response to immune challenge in *Eristalis*. Quantitative real-time PCR analyses revealed that transcripts of α-tubulin were reduced 18 fold, whereas transcripts of Et-AMP10 and eristalin were enriched for 4 and 38 fold, respectively (Fig. 2). This is in agreement with values from the protocols of the manufacturer and indicates successful subtraction of the cDNA library. A total of 288 clones were randomly picked and subjected to colony PCR. Plasmids of 117 colonies that were positively screened in blot hybridization, indicating induced expression in response to LPS challenge, were isolated and sequenced. The sequences obtained (summarized in table 1) were deposited at EMBL European Bioinformatics Institute, and compared to databases of the National Center for Biotechnology Information using the program BLASTX. InterProScan at the EMBL European Bioinformatics Institute was used for an integrated search in PROSITE, Pfam and PRINTS databases in order to predict conserved motifs, signal sequences and transmembrane regions.

**Table 1:** cDNAs from the subtracted *E. tenax *library

Cluster	EMBL accession No.	Highest BLASTX match	PFAM	*E *value
*Protein biosynthesis*				
	AM706409	Ribosomal protein S8 (*Culicoides sonorensis*)	PF01201	7e-09
	AM706410	Ribosomal protein L6 (*D. melanogaster*)	PF01159	3e-37
	AM706411	Lysyl-tRNA synthetase (*D. melanogaster*)	PF00152	6e-111
	AM706412	eIF5c (*D. melanogaster*)	PF02020	8e-84
*Repression of cell proliferation*				
	AM706413	CREG (*D. melanogaster*)		3e-46
*α-tubulin-like protein*				
	AM706414	AlphaTub85E (*D. melanogaster*)	PF03953	5e-18
*dsRNA-binding domain-like protein*				
	AM706415	Replicase polyprotein (*Drosophila C virus*)	PF00035	5e-05
*Pattern recognition protein*				
	AM706416	PGRP-SB1 (*D. melanogaster*)	PF01510	6e-64
*Signal transduction*				
	AM706417	Relish (*Glossina morsitans morsitans*)	PF00023	7e-35
	AM706418	Protein kinase (*D. melanogaster*)		5.2
*Kazal-type serine protease inhibitor*				
	AM706419	enhancer of split m1 protein (*D. simulans*)	PF00050	4e-07
*Putative antimicrobial peptides*				
Et-AMP1	AM706420	Defensin (*Phlebotomus duboscqi*)	PF01097	3e-12
Et-AMP2	AM706421	Bactenecin-7 (*Bos taurus*)		0.96
Et-AMP3	AM706422	Tachycitin (*Tachypleus tridentatus*)	PF01607	3e-06
Et-AMP4	AM706423	Salivary protein SG3 (*Anopheles stephensi*)		7e-15
Et-AMP5	AM706424	α-helical cecropin-like		NSM^1^
Et-AMP6	AM706425	Ser/Thr/Lys-rich putatively disulfide bridged		NSM
Et-AMP7	AM706426	Putatively disulfide bridged peptide with a p*I *10,5		NSM
Et-AMP8	AM706427	Putatively disulfide bridged peptide with a p*I *10,3		NSM
Et-AMP9	AM706428	Putatively disulfide bridged peptide with a p*I *9,3		NSM
Et-AMP10	AM706429	Putatively disulfide bridged anionic peptide		NSM
Et-AMP11	AM706430	Glycine-rich peptide 1		NSM
Et-AMP12	AM706431	Glycine-rich peptide 2		NSM
Et-AMP13	AM706432	Glycine-rich peptide 3		NSM
Et-AMP14	AM706433	Glycine-rich peptide 4		NSM
Et-AMP15	AM706434	Linear peptide 1		NSM
Et-AMP16	AM706435	Linear peptide 2		NSM
Et-AMP17	AM706436	Linear peptide 3		NSM
Et-AMP18	AM706437	Linear peptide 4		NSM
Et-AMP19	AM706438	Linear peptide 5		NSM

^1^NSM, no significant match

We describe here the identification of immune-inducible proteins in *E. tenax *that are potentially involved in recognition of microbes, immune-related signaling, and antimicrobial effector mechanisms.

### Sensing of infection

**Figure 3:**
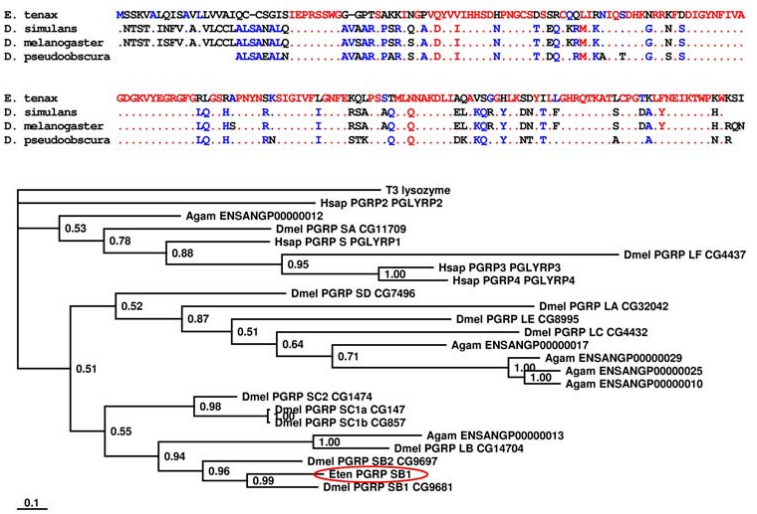
***E. tenax *PGRP is homologous to PGRP-SB1 from *Drosophila***. **(A) **BlastP search with *E. tenax *PGRP-SB1 sequence resulted in similar sequences from several *Drosophila *species and the differences are indicated in the alignment. Red color indicates 90% and blue color 50% consensus. The amino-terminal 24 amino acids represent a putative signal sequence. Accession numbers of PGRP-SB1 proteins are: *E. tenax*, AM706416; *D. simulans*, Q70PX8; *D. melanogaster*, Q70PY2; *D. pseudoobscura*, Q29DT4. **(B) **A Bayesian protein tree was generated using the sequence of *Eristalis *PGRP (Eten PGRP SB1, indicated by a red circle) and sequences of human (Hsap PGRPs), *D. melanogaster *(Dmel PGRPs), and *A. gambiae *PGRPs (Agam ENSANGPs) and we found that *Ersitalis *PGRP grouped nearest to *Drosophila *PGRP-SB1. T3 lysozyme (N-acetylmuramoyl-L-alanine amidase, P20331) of the bacteriophage T3 was used as out-group. The scale bar represents the substitutions per site according to the model of amino acid evolution applied.

Innate immunity depends on pathogen recognition that is mainly mediated by host proteins called pattern recognition receptors. In insects, two prominent members, the Gram-negative binding proteins (GNBP) and peptidoglycan recognition proteins (PGRP) are involved in the activation of prophenoloxidase cascade and of Toll/IMD pathways resulting in the massive production of immune effector molecules [6]. Here, we identified transcripts of a protein that shares sequence similarities with PGRP-SB1 from several *Drosophila *species (Fig. 3A). This peptidoglycan recognition protein (PGRP) is one of a battery of pattern recognition proteins in insects that are essential for recognition of invading microbes and induction of immune responses [14]. *Drosophila *and *Anopheles *have 13 and 7 PGRP genes that are transcribed into at least 17 and 9 proteins, respectively [14]. Mammals possess a family of 4 PGRPs that function as both recognition proteins and effector molecules exhibiting bactericidal activities [15]. A Bayesian protein tree (Fig. 3B) was generated with sequences from *Eristalis*, humans, *D. melanogaster*, and *A. gambiae *and revealed that *Eristalis *PGRP groups nearest to *Drosophila *PGRP-SB1.

### Signaling

We identified a protein sharing highest similarity with the relish protein of *Glossina morsitans morsitans *(Uniprot: AAZ91474). Relish or Nuclear factor (NF)-kappa-B p110 subunit is a key molecule of the Imd pathway and is phosphorylated by LPS-activated I-kappa-B kinase complex before being cleaved [16]. The Relish-p110 subunit is cleaved within seconds after immune challenge into Relish-p49 and Relish-p68 subunits. The latter translocates into the nucleus and activates the transcription of numerous immune-related genes [6]. The NF-kappa-B/I-kappa-B signaling pathways are evolutionarily well conserved and widely distributed among the animal kingdom [17].

Many serine proteinase inhibitors have evolved in vertebrates and invertebrates to regulate vital serine proteinase cascades. These contribute to melanization and host defense responses in insects [18,19] as well as to hemostasis, fibrinolysis, and complement system in vertebrates [20,21]. Here, we identified one proteinase inhibitor that belongs to kazal-type serine proteinase inhibitors. Kazal domains are characterized by a well-preserved amino acid sequence containing three disulfide bridges, and display varying specificity for serine proteinases [22].

One identified cDNA encodes a protein that exhibits highest sequence similarities to the replicase polyprotein of the *Drosophila C *virus. This virus belongs to the picornavirus family (enveloped single-stranded RNA viruses) that also includes the prominent polio and foot-and-mouth disease viruses [23]. However, it should be mentioned here that the similarities between the sequences depends mainly on a dsRNA-binding domain-like region. This region is also present in a variety of cellular RNA-binding proteins with different structures and diverse functions including e.g. the human interferon-induced protein kinase [24].

### Protein biosynthesis and cell proliferation

We identified several transcripts of factors potentially involved in *Eristalis *protein biosynthesis including ribosomal proteins S8 and L6, lysyl-tRNA synthetase, and the translation initiation factor eIF5c. This is reasonable, since the massive production of antimicrobial peptides upon immune challenge depends on the activation of the cellular translation machinery in cells and tissues of the immune system [9,13,25]. Nevertheless, one important study reported the antibacterial activity of ribosomal protein L1 fragment of *Helicobacter pylori *[26]. Additionally, Park *et al*. [27] found that lysyl-tRNA synthetase was secreted from intact human cells as a signaling molecule to trigger proinflammatory response by binding to macrophages and peripheral blood mononuclear cells to enhance their migration and the production of TNF-α. Since lysyl-tRNA synthetase is highly conserved in evolution it is possible that it also serves as a signaling molecule in insects.

One transcript shared high sequence similarities with the evolutionarily conserved cellular repressor of E1A-stimulated genes (CREG). A finely-tuned balance exists between cell proliferation, cell division arrest, and apoptosis in the vertebrate immune response, and this may also be true for insects. CREG is a secreted glycoprotein that inhibits cell proliferation and enhances cell differentiation [28,29]. In humans, CREG was shown to bind to the cation-independent mannose 6-phosphate/insulin-like growth factor II receptor, and this receptor has been shown to be required for CREG-induced growth suppression. In insects, CREG has been shown to be expressed in adult female salivary glands of *A. gambiae *[30].

We found one transcript that exhibits similarity to the carboxyl terminus of α-tubulin from other organisms. Although α-tubulin is a major constituent of microtubules and a well-known housekeeping gene, many genes and pseudogenes exist that form the α-tubulin superfamily [31]. Induction of α-tubulin-like mRNA after immune challenge has also been observed in leech [25], and another study demonstrated that the dengue virus 2 directly binds to a 48 kDa tubulin or tubulin-like protein of C6/36 mosquito cells [32]. These results suggest that at least some α-tubulin-like proteins may be involved in immune responses. However, in this study α-tubulin itself was depleted by the subtraction process (Fig. 2) suggesting that it is not immune-induced and the identified sequence may be derived from a pseudogene or related isoform.

### Effector molecules

**Figure 4:**
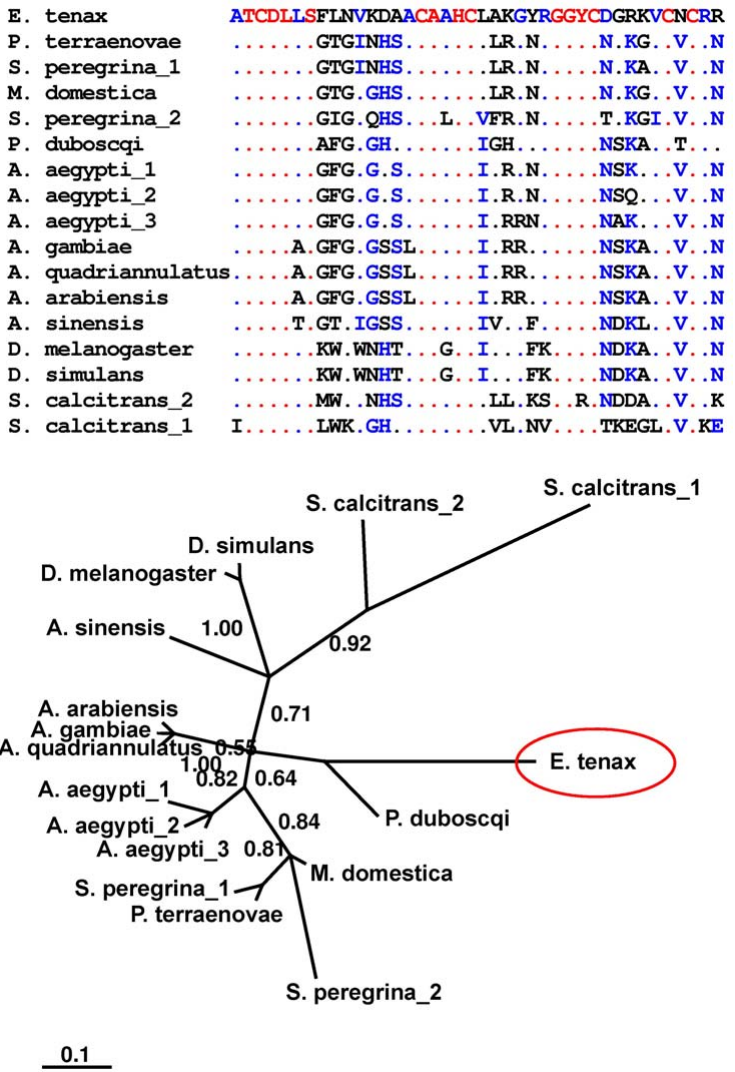
**Sequence alignment of eristalin with other dipteran defensins**. **(A) **Eristalin was aligned with other dipteran defensins. For clarity only sequences of mature defensins were used for alignment. Red color indicates 90% and blue color 50% consensus. **(B) **A Bayesian protein tree was generated and we found that *eristalin*, defensin_1 and defensin_2 of *Stomoxys calcitrans*, and defensin_1 of *Sarcophaga peregrina *show some higher deviation in sequence when compared to other dipteran defensins. Accession numbers of defensins are (from top to bottom): AM706420, P10891, P18313, Q86BU1, P31530, P83404, P91793, P81602, P81603, Q17027, Q38L94, Q38LE1, Q4ZJJ8, P36192, Q86BW0, O16137, O16136. The scale bar represents the substitutions per site according to the model of amino acid evolution applied.

The most striking finding among the genes inferred to be up-regulated in response to septic injury is the identification of numerous putative antimicrobial peptides in *E. tenax*. Defensins have been found in a variety of animals and plants [33-35] and even in the fungus *Pseudoplectania nigrella *[36], which is suggestive for an evolutionarily conserved role in innate immunity. In agreement, we identified an *E. tenax *defensin that we named eristalin. Eristalin contains a predicted signal and pro-sequence and shares sequence similarities to other insect defensins (Fig. 4A). In addition, our phylogenetic analysis using sequences of eristalin and other defensin sequences from dipterans suggests that the defensins of *E. tenax*, *S. peregrina*, and *S. calcitrans *are more deviated in sequence. Maggots of all three of this species live on feces (Fig. 4B) suggesting that independent occupation of such 'extreme' ecological niches is accompanied by divergent evolution of immune-related effector molecules.

**Figure 5:**
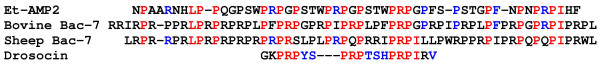
**Sequence alignment of Et-AMP2 with *Drosophila *drosocin along with bactenecin-7 from sheep and bovine**. For clarity only sequences of mature peptides of ET-AMP2 (AM706421), bactenecin-7 from sheep (UniProt: P50415) and bovine (UniProt: P19661), and of drosocin (UniProt: P36193) are shown. The predicted signal sequence of Et-AMP2 was omitted.

The putative antimicrobial peptide Et-AMP2 shares highest similarities to bactenecin-7 (Fig. 5) a cathelicidin-derived antimicrobial peptide of bovine neutrophil granules that exhibits antibacterial activity predominantly against gram-negative bacteria [37]. Bactenecin-7 belongs to the Pro/Arg-rich antibiotics [38] which also include porcine PR-39 [39], insect drosocin, abaecin, and lebocin [40]; however, no homologues were identified in humans. The antibacterial effects of Pro/Arg-rich peptides are likely due to an impaired function of the respiratory chain and of energy-dependent activities in the inner membrane of susceptible microorganisms [41].

ET-AMP3 shows high similarity to the antimicrobial tachycitin of the horseshoe crab [42] and ET-AMP4 to the salivary protein SG3, with as yet unknown function, from *Anopheles stephensi *[43]. Et-AMP5 is a cationic peptide with putative α-helical content and with similarities to cecropins or to antimicrobial cathelicidin-derived peptides like bovine BMAP-27 [44]. Et-AMP6, 7, 8, and 9 are putatively disulfide-bridged cationic peptides, while Et-AMP10 is a putatively disulfide bridged anionic peptide. None share similarities with any known proteins or peptides but all exhibit Lys/Arg clusters in the proximity of hydrophobic clusters; this is typical for many antimicrobial peptides [4].

**Figure 6:**
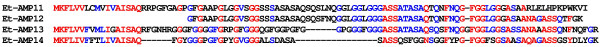
**Sequence alignment of the glycine-rich peptides Et-AMP11, 12, 13, and 14**. Sequences of the glycine-rich peptides Et-AMP11, 12, 13, and 14 (AM706430 to AM706433) were aligned. The alignment suggests that the isoforms have most probably been evolved by gene duplication and evolutionary selection to target a variety of pathogens. The amino-terminal 17 amino acids represent a predicted signal sequence. The amino-terminal end of Et-AMP12 is not complete.

**Figure 7:**
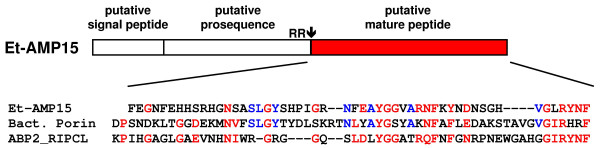
**Sequence alignment of the linear peptide Et-AMP15 with a bacterial porin and a putative antimicrobial peptide from *Riptortus clavatus***. The linear peptide Et-AMP15 (AM706434) is schematically drawn, showing predicted signal peptide, propeptide, and mature peptide. The mature peptide exhibits some sequence similarities with the carboxy-terminal part of bacterial porin from *Bordetella parapertussis *(Bact. Porin, UniProt: Q7W5A6) and a putative antimicrobial peptide from the bug *R. clavatus *(ABP2_RIPCL, UniProt: Q27906).

We also identified four isoforms (Et-AMP11 to 14) of glycine-rich peptides with a glycine content of over 20% (Fig. 6). The list of glycine-rich antimicrobial peptides is rapidly growing and includes prominent members like the antifungal holotricin-3 from the chafer *Holotrichia diomphalia *[45], the antibacterial acanthoscurrin from the spider *Acanthoscurria gomesiana *[46], and the antibacterial armadillidin from the crustacean *Armadillidium vulgare *[47]. Interestingly, the observation that several isoforms of a glycine-rich peptide are present in a single organism is in agreement with the identification of infection-induced glycine-rich peptides in *C. elegans *[48] and of putative glycine-rich attacins in the lepidopteran *Antherea mylitta *[49]. Similarly, we identified five isoforms (Et-AMP15 to 19) of a linear peptide that are cationic with a glycine content of over 10% and share sequence similarities to the carboxyterminal half of bacterial outer-membrane porins and to a putative antimicrobial peptide from the bug *Riptortus clavatus *[50] (Fig. 7).

### Quantitative real time RT-PCR analysis

**Figure 8:**
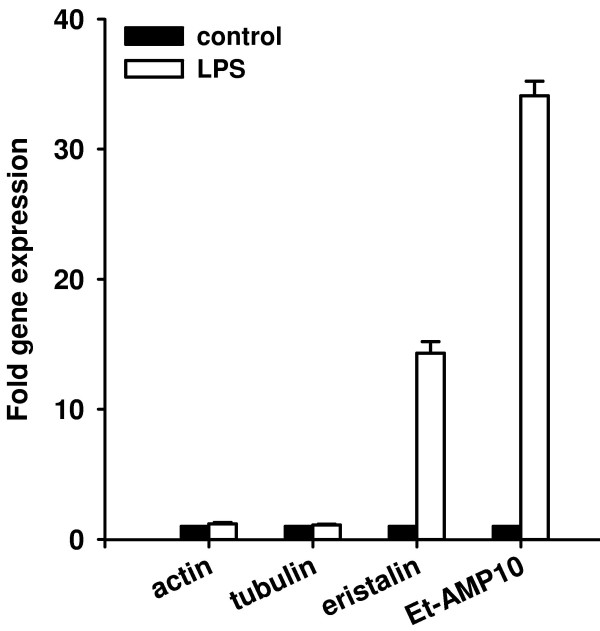
**Quantitative real time RT-PCR analysis of transcriptional levels of selected genes in immune challenged and untreated larvae**. The mRNA levels of selected genes in immune challenged animals (black bars) were determined and are shown relative to their expression levels in untreated animals (white bars). The transcriptional rates of eristalin and ET-AMP10 were found to be increased over 14 and 34 fold, respectively, in response to LPS injection. In contrast, the amount of actin and α-tubulin transcripts were not significantly influenced. Results represent mean values of three independent determinations ± S.D.

In order to precisely determine the immune-induction of genes identified in the present study we used quantitative real-time RT-PCR analysis. The *Eristalis *defensin eristalin and the putatively disulfide-bridged anionic peptide Et-AMP10 were 14 and 34 fold, respectively, up-regulated upon LPS injection (Fig. 8). The determined induction levels are in a similar range as reported for defensins from other insects [51]. In contrast, the expression of the housekeeping genes actin and tubulin were not influenced by the treatment.

## Conclusion

In the present study we analyzed the immune-inducible transcriptome from rat-tailed maggots of the drone fly *E. tenax *which have evolved the ability to survive in stagnant, frequently anaerobic aquatic habitats with extreme microbial contamination [3]. Survival in environments with such a strong microbial stress obviously depends on an innate immune system that has been adapted specifically to this ecological niche.

To date, seven distinct antimicrobial peptides have been reported from *Drosophila*, including the antifungal peptides drosomycin and metchnikowin, and five antibacterial peptides: attacin, cecropin, defensin, diptericin and drosocin [40]. Recently, genome-wide studies with *Drosophila*, *Anopheles*, and *Aedes *reveal that intracellular components of immune signaling pathways are conserved among Diptera [8]. In accordance, we identified potential counterparts in *E. tenax *that mediate non-self recognition (e.g. PGRP) or immune-related signaling (e.g. relish) in *Drosophila *and *Anopheles*. However, genome-wide studies also elucidate differences between both dipterans, particularly in potential extracellular components of the Toll pathway and in effector molecules including antimicrobial peptides [8]. In agreement, we identified numerous putative antimicrobial peptides in *E. tenax *that have obviously no homologues in *D. melanogaster*,*A. gambiae*, or *A. aegypti*. In total, we found 19 putative antimicrobial peptides some of which show sequence similarities to antimicrobial defensins, bactenecin-7, or tachycitin. 16 peptides are of novel-type including four isoforms of a glycine-rich peptide and five isoforms of a linear peptide. The isoforms may have been evolved by gene duplication and evolutionary selection to target a variety of pathogens, probably in a similar manner to that described for the six α-defensins and four β-defensins from humans [52].

Interestingly, we observed a significant *E. coli *inhibitory activity of hemolymph samples from untreated *E. tenax *larvae. This was not normally the case with hemolymph samples from other insect species that we analyzed in our laboratory (e.g. *Thermobia domestica *[9], *Galleria mellonella *[10], and *Tribolium castaneum*). This significant (probably constitutive) expression of antimicrobial peptides might be caused by the uptake of food with a high bacterial load. Recently, it has been demonstrated that *Drosophila *gut cells are able to sense bacteria-contaminated food which induces epithelial immune responses [6]. Thus, there may be an even richer assembly of immunity-related genes in *Eristalis *that would not be detected by our SSH approach.

Future work will include chemical or recombinant production of the identified peptides in order to analyze their specific activities against viruses, bacteria, fungi, or protozoa. Currently, we are investigating the potential of Et-AMP10 and eristalin to confer resistance against phytopathogens in transgenic plants similar as recently shown for the antifungal peptide gallerimycin from the greater wax moth *Galleria mellonella *[53]. In addition, the identified genes may help to elucidate divergent evolution of antimicrobial peptides in Diptera and the interdependence between ecological adaptation and insect immune defenses.

## Methods

### Insects

Rat-tailed maggots were collected in liquid manure pits at cattle farms near Giessen (Hessen) and the village Siewisch (Brandenburg), Germany. Using a practicable key for determination of hoverfly larvae [54], we identified third instar larvae of the drone fly *E. tenax*. As typical characters we observed the lack of setae along the lower lateral margins and the last pair of prolegs with most of the large primary crochets facing towards the lateral margins of the body. Identification was confirmed by determination of adults which hatched at the end of October 2005.

### Immune challenge of larvae and isolation of hemolymph and mRNA

Last instar *E. tenax *larvae, each weighing between 200–300 mg, were used for immune-challenge. 10 μl sample volume, corresponding to 100 μg LPS (purified *Escherichia coli *endotoxin 0111:B4, Cat. No.: L2630, Sigma, Taufkirchen, Germany) per larva were injected dorsolaterally into the hemocoel using 1 ml disposable syringes and 0.4 × 20 mm needles mounted on a microapplicator. For antibacterial activity assays, hemolyph samples were isolated by bleeding injected larvae 24 h post challenge or untreated larvae into plastic tubes. Total RNA was extracted from whole larvae 8 h post injection using the TriReagent isolation reagent (Molecular Research Centre, Cincinnati, Ohio, USA) and a further poly-(A)^+ ^RNA preparation was performed using a mRNA Nucleotrap kit (Macherey Nagel, Germany) according to the instructions of the manufacturers. RNA integrity was confirmed by ethidium bromide gel staining and quantities were determined spectrophotometrically [55].

### Antibacterial activity assay

Antibacterial activity of hemolymph samples was measured by an inhibition zone assay using a LPS-defective, streptomycin- and ampicillin-resistant mutant of *Escherichia coli *K12 strain D31 [56]. In brief, Petri dishes (∅ 100 mm) were filled with 7 ml *E. coli *suspension, containing 2× YT-nutrient broth (Roth, Karlsruhe, Germany), 1% high-purity agar-agar (Roth), and 2 × 10^4 ^viable bacteria in logarithmic growth phase. Holes with a diameter of 4 mm were punched into the agar and filled with 3 μl of cell-free hemolymph. The diameters of the clear zones were measured after 24 h of incubation at 37°C and units/ml were calculated using a calibration curve obtained with dilutions of gentamycin (Sigma).

### Construction of a subtracted cDNA library using the SSH method

In order to identify differentially expressed genes during immune response the SSH method was performed using mRNAs from immune challenged and control larvae and the PCR-Select cDNA Subtraction Kit from Clontech (Mountain View, CA, USA), according to the protocol of the manufacturer. Briefly, 1 μg of purified mRNA from immune-challenged and control larvae were reverse transcribed into cDNA using a cDNA synthesis primer, subsequently double stranded cDNA was generated and digested with *Rsa*I. The double stranded cDNA from immunized larvae was ligated in separate aliquots to adaptor 1 or adaptor 2R and were denaturated at 98°C for 90 s and then hybridized at 68°C for 8 h with a 30 fold excess of double stranded cDNA from control larvae. Subsequently, both samples were mixed together again with a 10 fold excess of freshly denaturated double stranded cDNA from control larvae and hybridized in one tube at 68°C for 16 h. The sample was then subjected to two rounds of suppression PCR with PCR-primer 1 and nested primers supplied with the kit. PCR amplifications were performed in a total volume of 25 μl using a PCR cycler (Biometra, Göttingen, Germany) with a heated lid and the Advantage PCR system (Promega, Mannheim, Germany). An initial adapter extension at 72°C for 5 min was followed by a denaturation step at 95°C for 1 min and by 27 cycles of denaturation at 95°C for 15 s, annealing at 66°C for 30 s, and extension at 72°C for 90 s. A final 7-min 72°C step was added to allow complete extension of the products. The secondary PCR was performed with nested primer 1 and 2R on the diluted primary PCR products for 12 cycles under same conditions, except that 68°C was used as annealing temperature. The subtraction efficiency was confirmed by quantitative real-time PCR of the actin gene of subtracted PCR products in comparison to not subtracted PCR products. Resulting PCR products of the secondary subtractive PCR were separated on 1% (w/v) agarose gel electrophoresis with ethidium bromide staining, according to standard procedures [55] and five fractions were obtained by excising five gel pieces of PCR products with different lengths. Subsequently, PCR product fractions were separately purified using the NucleoSpin Extract II kit (Macherey Nagel), ligated into pGEM-T easy vector (Promega) and transformed into TOP10F' cells (Invitrogen, Carlsbad, CA, USA). The library was plated on 2×YT agar plates containing 100 μg/ml ampicillin and incubated at 37°C for 16 h. A preliminary screen of 15 colonies using the FastPlasmid Mini kit (Eppendorf, Hamburg, Germany) followed by *Eco*RI digestion of isolated plasmids and agarose gel electrophoresis showed that over 90% of clones contained an insert in the vector.

### Colony PCR and blot hybridization

Colony PCR was performed on 288 randomly picked colonies with vector specific primers T7-promotor: 5'-TAATACGACTCACTATAGGG-3' and SP6: 5'-ATTTAGGTGACACTATAG-3' (purchased from Thermo electron, Waltham, MA, USA) using a Biometra PCR cycler and the Red Taq PCR system (Sigma, Taufkirchen, Germany). Used PCR conditions were: denaturation at 95°C for 3 min followed by 30 cycles of denaturation at 95°C for 15 s, annealing at 43°C for 15 s, and extension at 72°C for 60 s. A final 7-min 72°C step was added to allow complete extension of the products. 1 μl of resulting PCR products were identically spotted onto two sheets of positively charged nylon membranes (Roche, Lewes, United Kingdom). Membranes were dried and UV cross-linked using a BioRad UV cross-linker (BioRad, München, Germany), according to the instructions of the manufacturer. Digoxigenin labeled probes for hybridization were generated using secondary PCR products of subtracted and non-subtracted cDNAs and the Dig-High Prime Labelling kit (Roche, Lewes, United Kingdom). Hybridization, washing, and detection of digoxigenin labeled DNA was performed in accordance to the user guide instructions of the Dig Easy Hyb Granules, Dig-Wash and Block Buffer Set, Anti-Digoxigenin-AP and NBT/BCIP ready-to-use tablets (Roche).

### Sequencing and computer analysis of cDNA sequence data

Plasmid isolation of 117 positively screened colonies was performed with the FastPlasmid Mini kit (Eppendorf) and purified plasmids were custom sequenced by Macrogen Inc. (Seoul, South-Korea). Sequences were used to identify similar sequences of the National Center for Biotechnology Information databases using BLASTX program (BLASTX 2.2.13; http://www.ncbi.nlm.nih.gov/BLAST/). InterProScan http://www.ebi.ac.uk/InterProScan/ was used for an integrated search in PROSITE, Pfam, and PRINTS databases at EMBL-European Bioinformatics Institute and to predict signal sequences and transmembrane regions.

### Sequence alignments and phylogenic analyses

Sequence alignments were computed using the blosum62 algorithm [57] at http://bioinfo.genopole-toulouse.prd.fr. For phylogenetic reconstruction, we used the software package MrBayes 3.1.2 [58], which combines Bayesian inference and Markov chain Monte Carlo convergence acceleration techniques known as Metropolis coupling. The best fixed-rate model of amino acid evolution was determined by model jumping among nine possible models. The model with the overall highest posterior probability was WAG [59] for PGRPs and Blosum62 model [60] for defensins. Generations were sampled with the current tree saved at intervals of 100 generations. We used convergence diagnostic (i.e., the standard deviation of split frequencies) to determine whether the run length was sufficient. The average standard deviation of split frequencies at 2 × 10^6 ^generations was 0.0076 for PGRPs and at 10^7 ^generations 0.0023 for defensins, respectively. This therefore indicated that the two chains that were run converged on similar results. The 50% majority rule tree presented here was constructed from all sampled trees with the first 25% of all trees. Trees were visualized with TREEVIEW 1.6.6 [61]. Posterior probabilities plotted at the nodes can be interpreted as the probability that the tree or clade is correct [62].

### Quantitative real time RT-PCR

Quantitative PCR was performed with the real-time PCR system Mx3000P (Stratagene, La Jolla, California, USA) using the FullVelocity SYBR^® ^Green QRT-PCR Master Mix (Stratagene, La Jolla, California, USA), according to the protocols of the manufacturer. In order to confirm the subtraction efficiency of constructed cDNA library 1 ng of unsubtracted and subtracted cDNA, respectively, was used to amplify α-tubulin, Et-AMP10, and eristalin. Used primers were: the universal primers α-tubulin-forward: 5'-GCCAACCAGATGGTCAA-3' and α-tubulin-reverse: 5'-GCTTGGTCTTGATGGTG-3', eristalin-forward: 5-ATGGCTACATGTGATCTGCT-3', eristalin-reverse: 5'-ACGGCAATTGCAGACT-3', Et-AMP10-forward: 5'-ATGGACCCTCTTCTGTGG-3', Et-AMP10-reverse: 5'-TGGGCATCTGACAATA-3'. For gene expression analyses, we used 100 ng total RNA per well and primers described above. In addition, insect universal primers actin-forward: 5'-ATCCTCACCCTGAAGTACCC-3 and actin-reverse: 5-CCACACGCAGCTCATTGTA-3' were used to amplify *Eristalis *actin. Primers were selected using the primer3 software [63] and were purchased from Thermo electron (Waltham, MA, USA).

## Competing interests

The author(s) declare that they have no competing interests.

## Authors' contributions

BA designed and carried out the experiments, performed the analyses, and drafted parts of the manuscript. AV collected and immune challenged the animals, participated in the experimental design and coordination, and drafted parts of the manuscript. All authors read and approved the final manuscript.
